# Time‐efficient and flexible design of optimized multishell HARDI diffusion

**DOI:** 10.1002/mrm.26765

**Published:** 2017-05-30

**Authors:** Jana Hutter, J. Donald Tournier, Anthony N. Price, Lucilio Cordero‐Grande, Emer J. Hughes, Shaihan Malik, Johannes Steinweg, Matteo Bastiani, Stamatios N. Sotiropoulos, Saad Jbabdi, Jesper Andersson, A. David Edwards, Joseph V. Hajnal

**Affiliations:** ^1^ Centre for the Developing Brain King's College London London UK; ^2^ Biomedical Engineering Department King's College London London UK; ^3^ FMRIB Centre Oxford University Oxford UK; ^4^ School of Medicine University of Notthingham Notthingham UK

**Keywords:** magnetic resonance imaging, diffusion MRI, sequence development, neuroimaging, neonatal, developing brain

## Abstract

**Purpose:**

Advanced diffusion magnetic resonance imaging benefits from collecting as much data as is feasible but is highly sensitive to subject motion and the risk of data loss increases with longer acquisition times. Our purpose was to create a maximally time‐efficient and flexible diffusion acquisition capability with built‐in robustness to partially acquired or interrupted scans. Our framework has been developed for the developing Human Connectome Project, but different application domains are equally possible.

**Methods:**

Complete flexibility in the sampling of diffusion space combined with free choice of phase‐encode‐direction and the temporal ordering of the sampling scheme was developed taking into account motion robustness, internal consistency, and hardware limits. A split‐diffusion‐gradient preparation, multiband acceleration, and a restart capacity were added.

**Results:**

The framework was used to explore different parameters choices for the desired high angular resolution diffusion imaging diffusion sampling. For the developing Human Connectome Project, a high‐angular resolution, maximally time‐efficient (20 min) multishell protocol with 300 diffusion‐weighted volumes was acquired in >400 neonates. An optimal design of a high‐resolution (1.2 × 1.2 mm^2^) two‐shell acquisition with 54 diffusion weighted volumes was obtained using a split‐gradient design.

**Conclusion:**

The presented framework provides flexibility to generate time‐efficient and motion‐robust diffusion magnetic resonance imaging acquisitions taking into account hardware constraints that might otherwise result in sub‐optimal choices. Magn Reson Med 79:1276–1292, 2018. © 2017 The Authors Magnetic Resonance in Medicine published by Wiley Periodicals, Inc. on behalf of International Society for Magnetic Resonance in Medicine. This is an open access article under the terms of the Creative Commons Attribution License, which permits use, distribution and reproduction in any medium, provided the original work is properly cited.

## INTRODUCTION

Diffusion magnetic resonance imaging (dMRI) is able to provide insight into the complex neural fibre tract architecture of the human brain in vivo 1. It has clinical applications in the study, diagnosis and treatment of neurological disorders 2, as well as many scientifically focused applications, with an important emerging focus on building comprehensive models of human brain connectivity 3, 4. The acquired dMRI data, using specific gradients with varying magnitude and direction that introduce sensitivity to water diffusive processes of molecules in the brain, provide the input to the chosen analysis pipeline and thus determine and limit its capacity to probe structural complexity of the human brain. The potential of dMRI in this context continues to grow with advanced techniques such as biophysical modelling 5, 6, 7, 8, which allow more precise mapping of micro‐structure. However, these advanced analyses require ever more data, with current trends requiring not only the sampling of a large number of diffusion sensitization directions, such as in high angular resolution diffusion imaging (HARDI) 9, but also the collection of multiple *b*‐value shells 6, 7, 8. Furthermore, strategies such as diffusion spectrum imaging 10 focus on the whole diffusion sensitization space, constructed by diffusion directions and sensitization levels beyond the single spherical shell concept. It is clear that the growing range of diverse analysis models motivates a drive toward both increased time efficiency and flexibility regarding the choice and ordering of diffusion sensitization in the dMRI acquisition.

An ideal dMRI acquisition would provide whole brain coverage volumes with a high spatial resolution, combined with a completely flexible sampling of the diffusion sensitization space while producing data with high signal‐to‐noise ratio (SNR) at a maximum rate of data acquisition to allow as many independent samples as possible in a feasible examination time. However, both physiological and hardware constraints impose limits. Diffusion scans with the desired high sensitization (*b*‐value) push the boundaries of the capacities of modern gradient coil systems, with maximum gradient amplitude, slew rate and duty cycle (fraction of time for which demand can be placed on gradient system) all imposing limits on the acquisition. In addition to hardware constraints, there are also challenges associated with in vivo studies, such as limited scan time, subject motion, scan interruptions, and even early scan aborts 11, 12. To maximize utility, the acquisition needs to be carefully designed to be as time‐efficient and self‐consistent as possible and ideally to be robust to non‐ideal examination subjects and conditions. These requirements hold for any clinical cohort, but are particularly important for studies of neonates. The analysis of 52 dMRI scans from non‐sedated neonatal term subjects (gestational age 37.28–46.57 weeks, median 41.07 weeks) imaged in our institution in 2013 reveals that the acquisition was stopped before completion 17 times (33%) and required parts to be repeated in another 14 instances (27%) due to awakening or motion. Measures to accommodate this are thus beneficial for any study. The stimulus for this work is the developing Human Connectome Project (dHCP),^1^ aspiring to collect data from over 1000 newborns to gain insight into the development of the human brain. For the dHCP, the dMRI acquisition needs to be completed in approximately 20 min. The data will be shared with the scientific community and should thus be suitable for a wide range of processing algorithms and neuro‐developmental studies.

**Figure 1:**
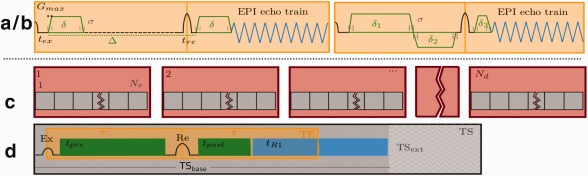
Two different diffusion preparations, the standard Stejskal‐Tanner (**a**) and the split diffusion gradient (SDG) preparation (**b**) are shown with the relevant diffusion parameters in green and the hardware‐set limits in black. The composition of the acquisition time of a dMRI scan with *N_d_* volumes using spin‐echo echo‐planar imaging is illustrated in (**c**) and (**d**). The number of volumes *N_d_*, each specifying a different given diffusion sensitization, influences the total number of TRs (c), (d) the TR itself depends on the length of the diffusion preparation shown in green, the length of the EPI read‐out shown in blue and the TS extension ${\text{TS}}_{\text{ext}}$ as resulting for example from the duty cycle.

With an acquisition time (TA) per slice shorter than 100 ms and tolerance to background phase variation caused by bulk motion 13, single‐shot spin‐echo echo‐planar imaging is still the most commonly used imaging method for dMRI. However, echo‐planar imaging (EPI) is sensitive to both T_2_ and $T_{2}^{*}$ decay. These constrain the echo time (TE) that can become long to achieve the chosen combination of *b*‐value and readout duration (Fig. 1a,b), and the duration of the readout which sets the vulnerability to signal dropout and susceptibility induced distortion in the phase encoding direction (PED). Numerous post‐processing techniques to correct for geometric distortions have been presented, such as the acquisition of additional *B*_0_ maps 14, correction using point‐spread‐functions 15 and the reversed gradient method 16. This last method generally employs a repeated acquisition of every diffusion sensitized slice stack with reversed PED to balance spatial collapse vs stretching, and uses an appropriate post‐processing technique to estimate and correct the underlying static magnetic field (*B*_0_). The approach guarantees recovery of the resolution through a least‐squares reconstruction at the cost of doubling TA. A technique combining all four PEDs was shown in 17 using retrospectively combined PEDs in combination with spatial and angular smoothness constraints.

Diffusion weighting of EPI sequences is commonly achieved by the Stejskal‐Tanner (ST) preparation of monopolar gradient lobes on either side of a single refocusing radiofrequency (RF) pulse 18 (Fig. 1a). There are variants including bipolar gradients 19 and the twice‐refocused diffusion preparation 20 that provide good eddy current properties at the expense of decreasing the efficiency of the preparation (i.e., prolonging it, tending to increase TE). A split diffusion gradient (SDG) approach (Fig. 1b), which splits the second diffusion gradient in two parts placed before and after the refocusing RF pulse to increase efficiency for a given *b* value and also to decrease the effect of eddy currents was proposed in simulations and in a small study by 21, 22. Recently, multiband (MB) imaging 23, 24 was used to accelerate the dMRI acquisition by acquiring multiple slices simultaneously 25, leading for example in the Human Connectome Project 26, 27 to a threefold acceleration. With acceleration comes an increased risk of spin history effects that can cause signal variation, particularly if there is subject motion, and consequently other sequence properties such as slice acquisition order can have increased importance 28.

These improvements and novel developments have all been realized individually for specific purposes. Achieving effective and efficient diffusion acquisitions that take advantage of the available approaches requires a number of disparate factors to be balanced, resulting in a complicated design problem. This work presents a comprehensive robust framework that combines key technical capabilities with an optimization process that addresses both gradient hardware constraints and vulnerability to interrupted or incomplete examinations. It is designed to acquire dMRI data with maximal time efficiency combined with flexibility to operate in different regimes where there are differing balances between conflicting goals and/or constraints and has the following key features:

- A MB capability combined with an optimized slice interleave pattern to accelerate and limit spin history effects.
- An optimized order and completely flexible sampling of diffusion encoding (*b*‐value shell and direction of sensitization within a shell) and PED, with option to freely mix all three in one single scan promoting consistency and usability of data acquired even in cases where sporadic movement and/or early scan termination may lead to data loss.
- A flexible diffusion preparation scheme that includes both ST and SDG patterns to allow TE minimization.
- A restart facility, allowing continuation of an interrupted scan to completion rather than requiring repetition from the start or a fixed break point.
- A thermal model that can be used to optimize the acquisition by adjusting all the above factors to minimize the impact of gradient duty cycle limits.

For thermal modelling, optimized order and slice acquisition order, scripts are provided in the additional material. The used symbols are found in Supporting Tables S2–S4.

In general, the inputs into these various optimization techniques depend on the study goals (these may fix the shells and ratios between shells), the population (which may define T_1_, T_2_, acceptable scan time and expected motion patterns), and the available hardware (described by *G*_max_, slew rate and thermal heating models). The framework was carefully validated and applied under different conditions, and then used to design two quite different protocols: a multishell HARDI protocol for use in the dHCP project and a higher spatial resolution shorter duration protocol for neurite orientation dispersion and density imaging (NODDI) 5 analysis. The approach and its ability to deliver input to different advanced analysis tools are demonstrated in the following sections.

## METHODS

The structure of the spin‐echo echo‐planar imaging diffusion weighted sequence is shown in Figure 1c as a combination of *N_d_* blocks (shown in red), each acquiring one complete slice stack (volume) for a specifically sensitized sample *d* in diffusion space. The acquisition of each volume, composed of *N_s_* slices, consists of acquiring *N_e_* ( $N_{e}\leq N_{s}$) shots of the base EPI sequence which takes a time TS_base_ per shot (in gray). In single‐shot acquisitions without the use of MB acceleration, the number of excitations equals the number of slices *N_e_* = *N_s_*. Figure 1d illustrates the spin echo timings for one TS_base,_ the time required for the read‐out illustrated in blue and the time available for diffusion preparation gradients in green. An additional time delay labelled TS_ext_ is shown, called TS extension, which may be required due to thermal heating of the gradient system (gradient coils or possibly the gradient amplifiers). The total slice time thus equals $\text{TS}={\text{TS}}_{\text{base}}+{\text{TS}}_{\text{ext}}$. The actual repetition time TR is calculated as $\text{TR}=N_{e}\cdot (\text{TS})$. Several mechanisms to reduce the total $\text{TA}=N_{d}\cdot N_{e}\cdot \text{TS}$ for a fixed number of diffusion samples *N_d_* were implemented and are presented in the following sections. These include the reduction of the number of shots using MB acceleration, reduction of the TS_ext_ by reordering to manage gradient demand and optimizations in the diffusion preparation.

### MB Acceleration

MB acceleration samples multiple slices at the same time and thus reduces the number of EPI shots per volume. With a MB factor of *N_m_*, $N_{e}=N_{s}/N_{m}$ excitations are required to achieve *N_s_* slices. MB excitation improves scan efficiency, but for constant bandwidth, the RF pulses amplitude increases at least as the square root of the number of simultaneous slices 26, 29, 30. To keep within the peak RF amplitude limit, it is necessary to stretch the pulses or even to time shift them 31 in order to keep the same bandwidth as a conventional pulse. Thus use of MB tends to increase the duration of the RF pulses, which increases TE.

A flexible MB scheme was implemented 32 allowing the number of slices and the field of view shift patterns used to distribute aliases according to the properties of the RF receiver coil array and the required field of view. MB excitation and refocusing pulses were carefully chosen to maximize slice excitation bandwidth and minimize necessary increase in TE; this was achieved using a combination of an asymmetric excitation pulse and a Gaussian refocusing pulse. Due to the use of Gaussian refocusing, time shifting of pulses was not beneficial 33. The optimal choice of the field of view (FOV) shift pattern depends on the properties of the coil and the coil geometry.

**Figure 2:**
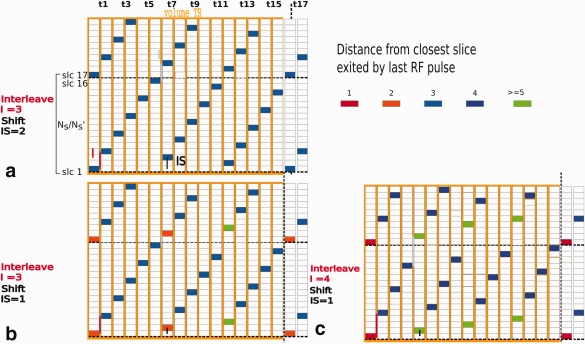
Illustration of the slice spacing and multiband acquisition order. The slice direction is shown vertically, the excitation order horizontally. The colors illustrate the spatial distance since the last excitation. Different combinations of interleave *I* and interleave shift IS are illustrated in (**a**), (**b**), and (**c**). The spatial distance (three slices) since last excitation is constant for (a) as it fulfills the requirements, whereas (b) and (c) show substantial deviations (ranging from gaps of one to five in different parts of the excitation pattern—see color code.

To allow for SNR enhancement, the slice thickness was chosen to be larger than the final target resolution. If the slice centers are located according to the desired target slice separation, resulting in overlapping slices, the target resolution can be partially recovered from the data using super‐resolution algorithms 34, 35 in a post‐acquisition reconstruction stage. To account for the option of overlapping slices and the expected sporadic subject motion, the slice interleave pattern is important. The scanner software was modified to provide maximal and constant temporal spacing between excitations in geometrically close slices, as well as across the boundaries between subsequent MB slice packs. One MB slice pack thereby specifies the slices between locations simultaneously excited from the same MB pulse 28 (Fig. 2).

This boundary condition depends on the number of excitations $N_{e}=N_{s}/N_{m}$, the interleave step *I*, defining the spatial distance between slices excited sequentially in time and the interleave shift IS, the spatial distance applied between the slices at the start of subsequent interleaves. The distance from the first slice in each interleave *i* to the slice pack start is given as $s_{i}=\;\mathrm{mod}\;((i-1)\text{IS},I)$. For each interleave *i*, the geometrical distance between the last excitation of this interleave and the first of the following must be equal to the interleave step distance *I* − 1. Thereby, the following constraint must hold true:

(1) $$\underset{\text{Distance}\;\text{last}\;\text{slice}\;\text{to}\;\text{slice}\;\text{pack}\;\text{end}}{\;\mathrm{mod}\;(N_{e}-(s_{i}+1),I)}+\underset{\text{Distance}\;1\text{st}\;\text{slice}\;\text{to}\;\text{slice}\;\text{pack}\;\text{start}}{\left(s_{i+1}\right)}=I-1\;\forall \;i\in [1,..,I].$$

This optimality condition is included into Supporting Script S1.

Single‐band volumes are acquired as a geometric reference and used together with a SENSE reference scan in the reconstruction pipeline for the MB images. The reconstruction is formulated as a linear inverse problem considering the coil sensitivities, the MB encoding pattern and EPI ghost correction terms. Reconstructed image and ghosting parameters are estimated from measured data in an alternating fashion 36 by using, respectively, the conjugate gradient algorithm and Newton's method 37.

### Flexible Sampling, Reordering, and Restart

To accommodate the needs of current and future multishell analysis techniques, it is necessary to allow complete flexibility in the distribution of the diffusion gradients 27. Even if such flexibility is supported by some vendors at least up to a certain extent, this does not reflect the general case. The sequence on our clinical 3T Philips Achieva scanner was therefore modified to allow arbitrary combinations and re‐ordering of diffusion gradient direction and magnitude for each acquired stack of EPI images. The complete freedom of choosing the diffusion weighting for every diffusion direction allows each shell to be optimized independently and all shells to be optimized together. An additional benefit comes from optimization of the temporal ordering, which can be used to 1 increase the motion tolerance of the complete dataset by ensuring that imaging volumes with similar diffusion sensitizations are widely separated in time of acquisition. This minimizes the risk that transient motion will cause data corruption over large contiguous regions of the diffusion sampling domain. 2 The option to interleave samples from different *b*‐shells and to select the order in which particular directions of sensitization are played out within a shell can be used to manage gradient demand (see below). 3 The temporal order can be chosen so that the angular density of coverage of each shell increases uniformly as data acquisition proceeds so that early termination leaves data that is most likely to be useful. To take advantage of situations where is it possible to continue after an interruption (e.g., after successfully resettling an infant), a restart facility has been implemented, which automatically records the last acquired diffusion sample and re‐starts the scan with a user specified number of volumes to be reacquired to compensate for motion just before the interruption.

### PED

**Figure 3:**
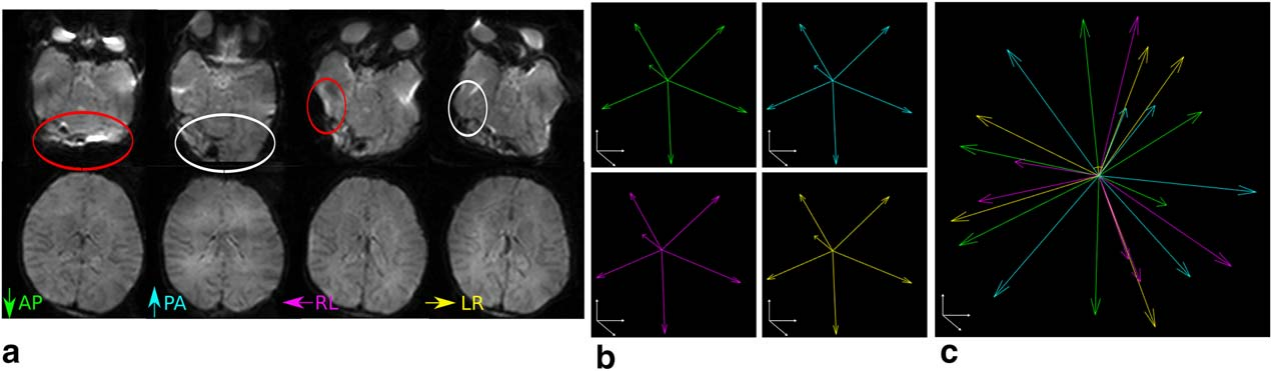
(**a**) Representation of two axial slices of a b0 volume in different PEDs. Complete double coverage of the sphere with all four PEDs and six directions in (**b**), (**c**) illustrates the joint interleaved PED acquisition using the same number of 24 diffusion directions.

The low bandwidth of EPI in the PED results in large spatial distortions. The choice of PED determines the direction of the distortions and whether this will be by stretching, which preserves pixel level information and so can be recovered by distortion correction algorithms, or spatial collapse, which can lead to irreversible information loss. Figure 3a, upper row, shows a transverse slice in a neonatal subject acquired in each of the four possible PEDs that can be selected for this slice plane. Examples of information loss by collapse are indicated by red ellipses in the anterior‐posterior (AP) and the right‐left (RL)‐PED slices, and complementary stretching can be observed in the PA and LR‐PED images (white ellipses). As the precise location and nature of distortions are highly subject and read‐out dependent, comprehensive protection against information loss due to voxel collapse requires complementary information. Advantages of acquiring images with all four PEDs over two PEDs have been recently shown 17 and the proposed acquisition has the potential to make use of this as well as any future method considering multiple PEDs.

In the following, two techniques will be discussed regardless of the number of PEDs used, the “separate” acquisition, which repeats all diffusion samples with reversed (or possibly all 4) PEDs, resulting in volumes jointly used for correction and the proposed “interleaved” technique, acquiring all PEDs in the same acquisition distributed over diffusion weightings without repetitions.

“Separate” comes with two drawbacks: limited self‐consistency (resulting from incomplete data due to early termination of scans or motion artefacts, and changes in subject position between repeats) and limited time‐efficiency, since the number of distinct angular samples is $N_{d}/2$ everywhere in the brain even though there are large regions that are not sufficiently impacted by susceptibility induced distortions to warrant this. An example is shown in Figure 3a, lower row, where in this more superior slice there is no significant collapse of information for any PED. In the “interleaved” case many voxels have up to *N_d_* samples and the voxels where resolution is lost due to distortion in certain PEDs still contain information from $\frac{1}{2}N_{d}$ or $\frac{3}{4}N_{d}$.

To provide maximum flexibility, the sequence was modified to allow independent selection of any PED for each diffusion sample, while enforcing consistent sequence timings and including safety checks for peripheral nerve stimulation, calculating the correct gradient duty cycle and including EPI ghosting correction data for all four PEDs. To explore the impact of this approach, a direct comparison was performed for the specific example of acquiring all four PEDs, but the general direction of improvements remains valid for the case of two PEDs. Figure 3b,c shows the distribution for a fixed number of *N_d_* = 24 diffusion directions acquired by repeating the same six unique directions in all four PEDs (b) and with interleaved PEDs (c). The nearest neighbor angle, used as a measure to assess the spherical density of diffusion samples, is reduced from 67.9° to 31.9°. To assess the strategy in practice, test data sets composed of *N_d_* = 28 diffusion directions (4*b* = 0, 24*b* = 2600) were acquired using (i) the full “interleaved” scheme and (ii) in four “separate” acquisitions using the same six non‐collinear directions in each PED. Acquisition (i) was repeated after (ii) to check the reproducibility of the results. Protocol **dHCP** was used for these experiments, the acquisition parameters can be found in Table 1.

**Table 1:** Acquired Experiments: (Left) dHCP Protocol, (Right) HighRes Protocol. Relevant Changes are Highlighted in Blue.

	dHCP protocol	HighRes protocol
Diffusion	*b* = 0 s/mm^2^ (20), *b* = 400 s/mm^2^ (64)	*b* = 0 s/mm^2^ (6), *b* = 750 s/mm^2^ (16), *b* = 2600 s/mm^2^ (32)
	*b* = 1000 s/mm^2^ (88), *b* = 2600 s/mm^2^ (128)	
	Stejskal‐Tanner Δ 42.5 ms, *δ* 14 ms	(a) Stejskal‐Tanner Δ 54.8 ms, *δ* 10.5 ms
		(b) Split diffusion *δ*_1_ 19.1 ms, *δ*_2_ 17.2 ms, *δ*_3_ 1.0 ms
Timing	TR 3800 ms TE 90 ms TA 19:20 min	(a) TR 3284 ms, TE 115 ms, TA 3:02 min
		(b) TR 7850ms TE 98, TA 7:13
	Echo spacing 0.81 ms, EPI factor 83	Echo spacing 0.911 ms, EPI factor 125
Geometry	FOV 150 × 150 × 102 mm^3^	FOV 150 × 150 × 102 mm^3^
	64 slices, slice overlap 1.5 mm	64 slices, slice overlap 1.5 mm
	Resolution 1.5 × 1.5 × 3 mm^3^	Resolution 1.2 × 1.2 × 3 mm^3^
Acceleration	Multiband 4, shift FOV/3	Multiband 4, shift FOV/3
	Interleave 3, interleave shift 2	Interleave 3, interleave shift 2
	SENSE 1.2, Partial Fourier 0.855	SENSE 1.2, Partial Fourier 0.855
Hardware	*G*_max_ 70 mT/m	*G*_max_ 80 mT/m
Acquired on	400 neonates	10 neonates

### Thermal Modelling

The heat generated in the gradient system (gradient coils and amplifiers) depends on the currents demanded to achieve the gradient waveforms (*G*) used by the sequences. Scans that impose high demands on the gradient system such as dMRI may require cooling periods to be inserted between successive repeats of the core sequence to avoid overheating that could cause damage. Thus, TR consists of the minimal required TS_base_ for individual EPI slice excitations, which is independent of thermal heating and these cooling periods, referred to as TS extensions (TS_ext_). The maximum temperature may occur at any time during the acquisition and may reach critical levels only for brief periods. However, to ensure uniform contrast properties, the TR must be the same for all slice stacks, so the most extreme TS_ext_ required to avoid overheating sets the minimum TR for the whole acquisition. The worst case thus determines the total time of the scan. For dMRI, the heating is dominated by the diffusion gradient lobes, although long EPI readouts can also impose significant demands. Optimization of the acquisition order within a given diffusion sampling scheme, can be used to control and mitigate the duration of TS_ext_ required to keep within hardware operation limits. The readout demand differs with the PED, so that flexible combination of diffusion sensitization and PED can add a small additional gain.

#### Heat Dissipation per Diffusion Direction

The acquisition consists of *N_d_* diffusion samples, where every *d_j_* is characterized by *b*‐value and *b*‐vector, which are translated into different waveforms: $G_{i}^{j}$ (axis i∈{x,y,z}), depending on the chosen diffusion preparation (see “Diffusion Preparation” section). These are combined with the imaging gradients (EPI read‐out gradients, slice re winders and auxiliary preparation gradients), which remain unchanged for all diffusion samples but vary with chosen PED. To achieve optimized sampling it can be helpful to match choices of PED to the pattern of diffusion gradients, and so in the following each *d_j_* with its associated waveform $G_{i}^{j}$ will include *b*‐value, *b*‐vector and PED information.

As all *N_e_* shots of every diffusion sampling are acquired consecutively in the time $\text{TR}=({\text{TS}}_{\text{base}}+{\text{TS}}_{\text{ext}})\cdot N_{e}$, the thermal development will be evaluated for each of these complete volumes only. In practice, multiple thermal factors, time constants *τ* and positions within the gradient coil are considered 38 which are hardware and thus vendor specific.

#### Cumulative Heat Effects

The temperature evolution is cumulative and evaluated over discretized time segments indexed by m=0,1,… with length *t_m_* ( ∼10 ms). We consider the dissipated power $P_{i}^{m}$ (axis i∈{x,y,z}) in [Watts = VA] generated in time segment *m*, calculated from the square of the gradient strength $G_{i}^{m}$ in (T/m) and an axis specific hardware factor, *H_i_*, which is the product of the square of the inverse of the gradient coil sensitivity in [T/m/A] and the coil resistance in [Ohms] = [*V/A*], and thus has units [ $VAm^{2}/T^{2}$]:

(2) $$P_{i}^{m}={\left(G_{i}^{m}\right)}^{2}H_{i}.$$

The combined thermal load $L_{i}^{m}$ [W] at time index *m* is calculated as the sum from the decaying previous thermal load (time point *m* − 1) and the generated thermal load in the current segment:

(3) $$L_{i}^{m}=\underset{\text{Previous decaying thermal load}}{L_{i}^{m-1}\exp(\frac{-t_{m}}{\tau })}+\underset{\text{Dissipated}\;\text{power}\;(\text{generated}\;-\;\text{decayed})}{P_{i}^{m}(1-\exp(\frac{-t_{m}}{\tau }))}.$$

Considering a specific set $D=\{d_{j}|\forall j=1,..N_{d}\}$ of diffusion samplings, where every *d* specifies a concrete set of diffusion parameters for a bval/bvec combination, the combined thermal load after sample *d_j_* equals $L_{i}^{j}$. It equals to $L_{i}^{m}$ obtained cumulatively with Equation (3), where *m* corresponds to the time index $\frac{j\cdot TR}{t_{m}}$. In the final step, the worst case over time (parametrized by diffusion sample *d_j_*) and gradient axes (*i*) is calculated $L=\max_{j,i}(L_{i}^{j})$. This value must never exceed a manufacturer specified hardware load limit, *L*_Hmax._ Should *L* get too large, the length of TS_ext,_ during which there is no gradient activity so that $P_{i}^{m}$ in Equation (3) is zero, can be increased to allow the thermal load to decay further in each TR. The whole calculation process can simply be repeated until a value of TS_ext_ is found for which *L* is always just less than *L*_Hmax_ for all *d_j_*.

#### Optimization Algorithm

Permutations of set *D*, specifying the order of its elements, are from now on referred to as Π(D) and element *j* in the permutation is referred to by *d_j_*. A constructive algorithm was implemented (and is detailed in Algorithm 1 below) to select the optimal order in terms of minimal thermal heating for a given set of *N_d_* samples of diffusion sensitivity space. There is no specific constraint for the set of *N_d_* samples, every sample can be chosen individually as given by the needs of the selected diffusion analysis model. The algorithm chooses in each iteration *y* a maximal target load $L_{\text{max}}^{y}$ and constructs a permutation $\Pi^{j}$ for every step *j* ( $j=1,..,N_{d}$) as long as a valid solution for the next time step exists. The next diffusion sampling is chosen among the remaining subset of as yet unused diffusion samples $D^{j}=\{d_{n}\forall n\in \{1,..,N_{d}\}\},d_{n}\notin \Pi^{j}\}$. The thermal load at the end of the current considered ${\text{TR}}_{\text{ext}}$ (as obtained from the current $L^{j+1}$) is calculated exhaustively per axis for all remaining diffusion weightings individually using Equation (3) with initial thermal load set to *L^j^*, the value from the previously chosen diffusion weighting. The maximum over all gradient directions *i* is chosen as $L_{n}^{j+1}=\max_{i}L_{i,n}^{j+1}$ and this must not exceed the hardware load limit, *L*_Hmax._ The next sample ( $d^{j+1}$) is thus chosen as

(4) $$d^{j+1}=\underset{{}_{D}}{\arg\max}(L_{n}^{j+1})\;\text{such}\;\text{that}\;\max((L_{d}^{j+1}))\leq L_{\text{max}}^{y}.$$

This process is repeated with maximal allowed load $L_{\text{max}}^{y}$. Supporting Script S2 provides implementation details.

Note that we deploy this model to determine the thermal load of the diffusion sequence in isolation without consideration of any prior gradient activity. This is actually a practically reasonable approach as sequence preparation and idle time is likely to allow heating from previously run sequences to be dissipated.

**Table:** 

Algorithm 1 Thermal optimization algorithm.
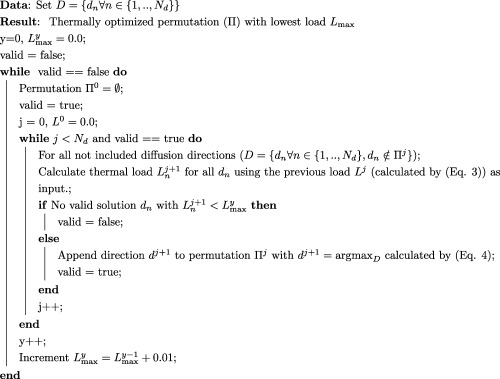

The input for the algorithm is, as stated above, a spherically optimized set of diffusion directions *D*. This set is generated for a multishell HARDI acquisition over all shells, following specification of each shell's *b*‐value and number of distinct directions, using electrostatic repulsion 39. The full set of sampling points is then split into four subsets (one per PED) with maximal intra‐subset electrostatic repulsion 11, 12, 40. The sequentially used optimization steps are specified in the paragraph “Ordering” in the Results section.

### Diffusion Preparation

The minimal achievable TE for a diffusion EPI sequence is determined by maximum *b*‐value required, hardware properties and physiological limits. The hardware settings may be specified by maximal gradient strength *G*_max_, maximal gradient slew rate, which together fix the minimal slope duration *σ*. On our scanner, a slew rate of 100 T/m/s was used, *σ* is obtained as $\sigma =\frac{G_{\text{max}}}{\text{slew}}$. Together with times *t*_ex_ and *t*_re_ required for the excitation and refocusing pulses respectively, these may be summarized by a vector $\vec{h}=(t_{\text{ex}},t_{\text{re}},G_{\text{max}},\sigma )$. The temporal efficiency of the diffusion weighted EPI is then critically dependent on the duration of the EPI readout required to achieve the desired image resolution. Specifically the length of the readout section that precedes the TE, $t_{R1}$, as illustrated in Figure 1d determines the available time for the diffusion gradients to be played out after the refocusing RF pulse. Because the time structure of the diffusion gradients must be the same on all axes, it is sufficient to analyze the diffusion sensitization along the read‐out axis *m* only. Figure 1a illustrates a typical EPI sequence diagram with a ST diffusion preparation. The diffusion properties are characterized by the duration of the diffusion gradients *δ* and the time between gradient pulses Δ: ${\vec{d}}_{\text{ST}}=(\delta ,\Delta )$. The *b*‐value for the ST preparation is calculated 21 as

(5) $$b(\vec{h},{\vec{d}}_{\text{ST}})=\gamma^{2}G^{2}(\delta^{2}(\Delta -\delta /3)-\sigma^{2}\delta /6+\sigma^{3}/30).$$

Keeping the same readout and diffusion weighting, the TE can be reduced by moving part of the second gradient lobe before the refocusing RF pulse, since the available time between excitation and refocusing pulses *t*_pre_ is longer than the time between the refocusing pulse and the beginning of the read‐out (*t*_post_) as shown in Figure 1a,d. Assuming a symmetric excitation pulse, the relation $\text{TE}=0.5\cdot t_{\text{ex}}+t_{\text{pre}}+t_{\text{re}}+t_{\text{post}}+t_{R1}$ holds.

This SDG configuration 21, 22 is illustrated in Figure 1b. The diffusion gradient lobes are conveniently parametrized by *δ*_2_ as the length of the second diffusion gradient and $r=\frac{\delta_{2}}{\delta_{3}}$ describing the ratio between second and third lobe durations, with $\delta_{1}=\delta_{2}+\delta_{3}$. Thereby, using the conventions in 21, *δ*_1_ includes 2 ramp times *σ* whereas $\delta_{2},\delta_{3}$ include 1 ramp time *σ*, the full length of the gradient objects is thus given by $\delta_{1},\delta_{2}+\sigma ,\delta_{3}+\sigma$. Assuming complete use of the available time *t*_pre_, the preparation is uniquely described by ${\vec{d}}_{\text{SDG}}=(\delta_{2},r)$ and the *b*‐value for this SDG preparation calculated as 21

(6) $$b(\vec{h},{\vec{d}}_{\text{SDG}})=\gamma^{2}G^{2}({\left(\delta_{2}(1+r)\right)}^{2}(t_{\text{pre}}-\delta_{2}-(\delta_{2}(1+r))/3)+{\left(r\delta_{2}\right)}^{2}(t_{\text{re}}+\sigma )-(1/6)(\delta_{2}(1+r))\sigma^{2}+(1/20)\sigma^{3}).$$

The practical implementation of the diffusion sequence calculation within the scanner software occurs in two steps and is depicted in Algorithm 2.

Step 1: Different TE settings using different combinations of imaging gradient timings and pulses, as specified by the vendor are proposed, each leading to available times *t*_pre_ and *t*_post_.

Step 2: For each of these settings, the diffusion gradients are included and the required $G_{\max}$ calculated, resulting either in a valid or invalid solution. For the SDG preparation, the maximum achievable *b*‐value $\tilde{b}$ for the current settings, is calculated using Equation (6) with the following settings:

(7) $$\delta_{3}=t_{\text{post}}-\sigma ,\;\delta_{2}=\delta_{3},\;\delta_{1}=t_{\text{pre}}-\delta_{2}-\sigma .$$

If $\tilde{b}<b$, no valid solution is possible and *G* is set to $G=G_{\text{over}}$, with $G_{\text{over}}>G_{\max}$. Otherwise, if $\tilde{b}\geq b$, a valid solution can be reached and the corresponding required *G* is calculated by minimizing TE within the given frame taking hardware limitations and the minimal crushing area *A* for sufficient FID dephasing after the refocusing pulse into account:

(8) $$\delta_{2},r=\arg\underset{\delta_{2},r}{\min}(f1,f2)\;\text{such}\;\text{that}\{\begin{array}{c} \delta_{2}/r+\sigma <t_{\text{post}},\;\delta_{2}+\delta_{2}/r+\sigma <t_{\text{pre}}\;(c1),\\ \delta_{2}/r+\sigma >A/G\;(c2)\;\text{and}\;\\ \delta_{2}>\sigma \;(c3). \end{array}$$

Thereby, the functions **f1**, **f2** are defined as follows:

(9) $$f1(\delta_{2},r)=\underset{\text{Residual}\;\text{between}\;\text{desired}\;\text{and}\;\text{achieved}\;b‐\text{value}}{\underset{︸}{\left|b(\vec{h},\delta_{2},r)-\hat{b}\right|}}$$

(10) $$f2(\delta_{2},r)=\underset{\text{Available}\;\text{time}\;\text{between}\;\text{pulses}}{\underset{︸}{t_{\text{pre}}}}-\underset{1\text{st}\;\text{diffusion}\;\text{lobe}}{\underset{︸}{\left(\delta_{2}(1+r)+2\sigma \right)}}-\underset{2\text{nd}\;\text{diffusion}\;\text{lobe}}{\underset{︸}{\left(\delta_{2}+\sigma \right)}}.$$

Constraint (**c1**) ensures that the diffusion gradients fit into the calculated sequence structure in step 1 and (**c2**) ensures that sufficient crushing after the refocusing pulse is achieved by forcing the area of the third lope to be greater than *A*/*G*. Constraint (**c3**) forces the second diffusion lobe to be bigger than *σ*. If is was smaller, the use of the SDG technique would not reduce the TE compared to the standard ST preparation and thus not be beneficial. The function (**f1**) minimizes the residual between desired and achieved *b*‐value, effectively assuring that the sequence produces the diffusion contrast the user asked for. Function (**f2**) assures efficiency, by using the additional degree of freedom given with SDG (ratio r) to minimize the empty time between the excitation and refocusing pulse and thus assuring that minimal TE is achieved. These functions are added with equal weight for the objective function of the optimization.

**Table:** 

Algorithm 2 Diffusion timing calculation including the SDG case.
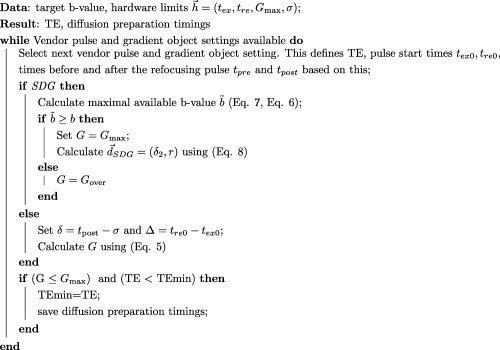

### Final Optimization

The elements described are combined together to optimize the dMRI acquisition given the population, study and hardware specific settings. The final step constitutes the choice of the diffusion preparation and the maximal gradient strength, chosen to achieve high signal. The signal‐to‐noise‐ratio SNR for the acquisition depends on the echo time TE, the TA, T_1_, T_2_ and the number of diffusion directions *N_d_* and is derived in the following:

Starting from the SNR equation for fixed slice thickness (neglecting details of the slice profile etc.), with *N* being the noise standard deviation,

(11) $$\text{SNR}=\frac{\rho \exp^{-\frac{\text{TE}}{T_{2}}}(1-\exp^{-\frac{\text{TR}}{T_{1}}})}{N},$$

the SNR for a dMRI acquisition with fixed TA, leading to the relation $\text{TR}=\text{TA}/N_{d}$, and assuming dense sampling 41, is given as

(12) $$\text{SNR}/\text{TA}=\frac{\rho }{\text{TA}\cdot N}\sqrt{N_{d}}\exp^{-\frac{\text{TE}}{T_{2}}}(1-\exp^{-\frac{\left(\text{TA}/N_{d}\right)}{T_{1}}}).$$

And, given fixed TA and noise level *N*, the derived SNR/TA equation is proportional to

(13) $$S:=\text{SNR}/\text{TA}\propto \sqrt{N_{d}}\exp^{-\frac{\text{TE}}{T_{2}}}(1-\exp^{-\frac{\left(\text{TA}/N_{d}\right)}{T_{1}}}).$$

Given a limited TA and known $T_{1}$ and $T_{2}$ 42, the optimization of the signal depends on TE and *N_d_*. TE can be decreased by increasing the maximum gradient strength $G_{\max}$ and choosing the diffusion preparation, parametrized by $\vec{d}$ (described in section Diffusion preparation for both ST and SDG preparation), whereas *N_d_* depends on thermal loading via the TS_ext_ which in turn depends on the gradient waveform details and may be highly sensitive to increasing $G_{\max}$. Thereby, for this case of fixed TA, both TE and *N_d_* depend uniquely on the diffusion variables and the maximum gradient strength $G_{\max}$:

(14) $$(G_{\max},\vec{d})=\underset{{}_{G_{\max},\vec{d}}}{\arg\max}S(\text{TE}(G_{\max},\vec{d}),N_{d}(G_{\max},\vec{d}):\text{TA},T_{1}{,T}_{2}).$$

The optimality algorithm thus takes the individual study requirements into account.

## RESULTS

The created modified dMRI acquisition is highly flexible and can be used to design quite different acquisitions depending on priorities and hardware constraints. It has been used to create an optimized acquisition for the dHCP project and this can serve to illustrate some key trade‐offs that the approach allows and can exploit. Key design requirements for dHCP are: total TA of 20 min, minimal imaged volume for the target population set at 145 × 120 × 96 mm^3^ which is sufficient for up to the 95th percentile for head size at 44 weeks gestational age, and a HARDI prescription having four shells (b0, b400, b1000, b2600) with a distribution of 5:16:22:32 volumes per shell that was optimized for the neonatal brain 43, 44. The second experiment was designed to optimally depict the cortical surface and thickness especially in pre‐term neonates. Therefore, a NODDI type protocol 5 with two shells, b750 (16 directions) and b2600 (32 directions) was designed with a focus on high spatial resolution and high SNR but without the limitation of a fixed TA as in the case of the **dHCP** experiment.

It makes furthermore use of the flexible PED capacities, as its 6 *b*‐zero volumes are distributed throughout the scan in pairs with reversed PED to allow for top‐up distortion correction. The optimized sequence has been implemented on a clinical 3T Philips Achieva system operating at up to 80 mT/m on each axis and was tested using phantoms, adults scanned in a standard Philips 32‐channel head coil and neonates imaged during natural sleep using a dedicated 32‐channel neonatal head coil (RAPID Biomedical, Rimpar, Germany) and patient handling system 45. Written, informed consent was obtained for all participants prior to scanning. Neonatal informed consent was provided by someone with parental responsibility. All study procedures were reviewed and approved by the Riverside Research Ethics Committee (14/LO/1169). The final optimized protocol parameters are summarized in Table 1. All images were reconstructed using the MB reconstruction described above and distortion corrected using FSL5's top‐up 16 and eddy 46.

### Exploring Diffusion Gradient Patterns to Minimize TE

**Figure 4:**
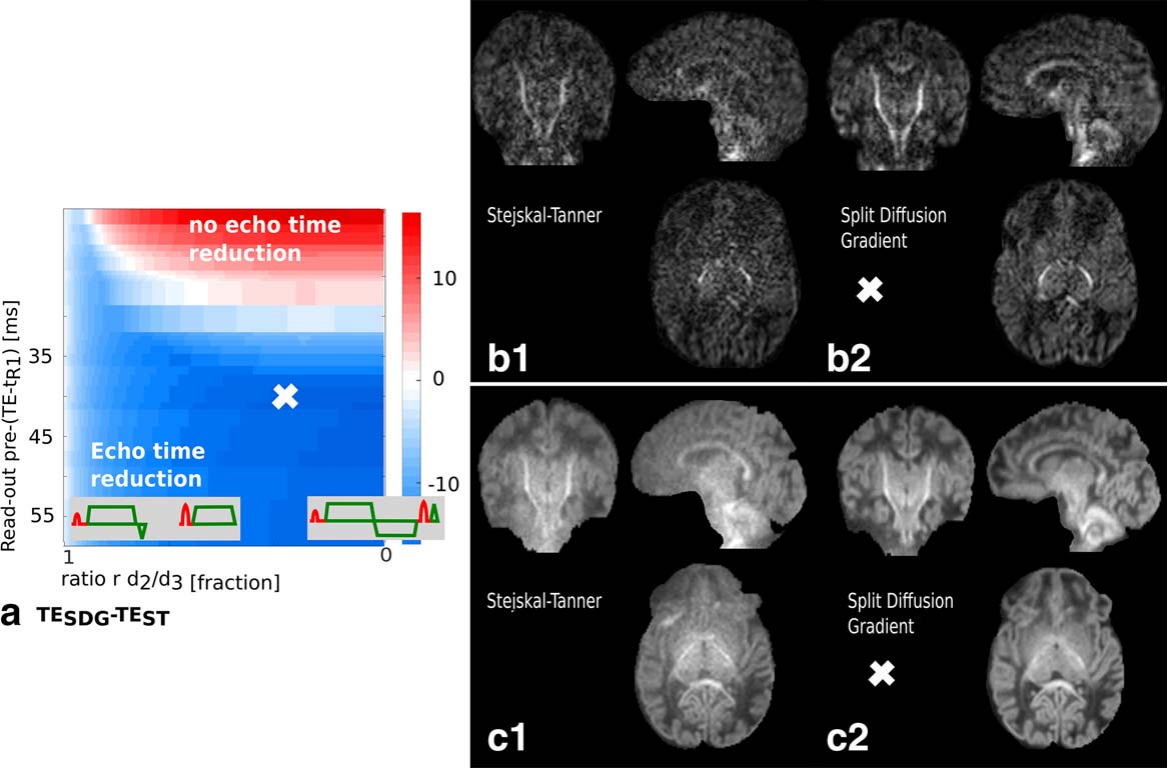
Results for the **HighRes** experiment. (**a**) The difference in TE between SDG and ST preparation for different read‐out length (as resulting from choices of SENSE factors of 1.0–2.0 and partial Fourier factors of 1–0.8) and ratios *r*. The chosen parameter setting for the high spatial resolution protocol is marked with a cross. Diffusion weighted *b* = 2600 for ST (**b1**) and SDG (**b2**) and mean *b* = 2600 diffusion weighted (DW) signal for ST (**c1**) and SDG (**c2**) from 30 volumes of the same subject.

A key parameter that impacts on SNR is TE and this can potentially be reduced for a given spatial resolution and diffusion weighting by adopting the SDG waveform. The limiting condition is always the maximum diffusion weighting, so we limit the following discussion to considering the maximum *b* value only, which in this case is 2600 s/mm^2^. The key sequence parameters to be considered are the duration of readout before the time of the spin echo and the ratio *r* between gradient lobe durations *δ*_2_ and *δ*_3_. Figure 4a shows the TE reduction ( ${\text{TE}}_{\text{SDG}}-{\text{TE}}_{\text{ST}}$) achieved using the SDG preparation with the maximum available gradient strength of 80 mT/m, a matrix size of 124 × 125 and different choices of SENSE (1.0–2.0), partial Fourier (1–0.8) and ratio *r* between *δ*_2_ and *δ*_3_. It shows that a beneficial setting is achievable for all read‐out lengths and there is an optimal *r*. Certain settings of *r*, corresponding to an extension in the spacing between excitation and refocusing, lead to an increased TE using SDG preparation, as indicated in the upper right corner of Figure 4a (shaded red). Thus, although there is always an optimal *r*, for a short readout the SDG pattern can result in longer TE than the ST preparation because of the constraints on the minimum duration of the second diffusion lobe. For these parameters, SDG is not beneficial.

For the given *b*‐value and gradient strength, the achievable reduction in TE reaches its peak for read‐out length of approximately 49 ms with a reduction of 19 ms, after which the crushing constraint limits further gains by inhibiting any further reduction in *d*_3_. The spatial resolution that can actually be achieved then depends on choices of parallel imaging (SENSE/GRAPPA) and partial Fourier factors.

**Figure 5:**
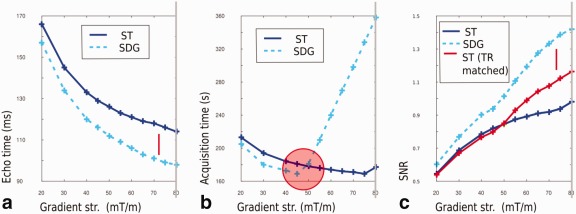
Results for the high resolution **(HighRes)** experiment with the chosen configuration marked with a gray line (here corresponding to Gradient strength = 80 mT/m). (**a, b**) The TE reduction and the TA is shown for different $G_{\text{max}}$. (**c**) The SNR per unit time for ST, SDG and TR matched ST is illustrated. The red line (TR matched) corresponds to a setting using the ST preparation with the TR matched to the SDG preparation. This version differs from the hardware optimized ST preparation (blue curve) only by its increased TR, which increases SNR by dint of making the scan take longer, although the result still remains inferior to the presented SDG preparation (light blue curve).

Although the benefit of using the SDG preparation increases with the duration of readout, so does the absolute TE. To explore key trade‐offs in the optimization, we set the readout duration to 90 ms which achieves an TE reduction from 115 to 98 ms when $G_{\text{max}}=80\;\text{mT}/m$. For fixed readout duration, the TE decreases with growing $G_{\text{max}}$. The duty cycle induced extension in TS, and thus the total imaging time TA, depends critically on the number of samples on the high *b* shell (Fig. 5a). For 32 directions, as in a conventional HARDI acquisition, TS_ext_ increases rapidly for the SDG preparation for gradient strength above $G_{\text{max}}=45\;\text{mT}/m$ due to excessive thermal demand (Fig. 5b). Since in this case the design did not include a total TA constraint, the curves in (Fig. 5c) (light blue for SDG and blue for ST) show the obtained SNR for the most efficient operating point given hardware constraints. There is a clear benefit for the SDG preparation despite the increase in TR compared to the ST preparation. The red line shows the SNR for the ST preparation with TR increased to match the SDG preparation to eliminate effects due to the longer TR. This demonstrates that although increasing the total TA for the ST preparation does increase its SNR, it is still less efficient then the SDG preparation in this case.

Predictions for a two‐shell protocol described above using a MB factor of 4, no SENSE and partial Fourier of 0.85 were verified with the ST preparation **HighRes‐a**: (TE/TR = 115/3284, TA 3:02) and the SDG preparation **HighRes‐b**: (TE/TR =98/7850 ms, TA = 7:13). The increase in TR for **HighRes‐b** compared to **HighRes‐a** is a result of the increased TS extension due to thermal heating. The cross in Figure 4a marks these imaging parameters. Figure 4b shows *b* = 2600 s/mm^2^ data acquired from the same exemplary subject using the ST preparation (b1) vs. the SDG preparation (b2). The mean diffusion signal calculated for both are shown in Figure 4 (c1) and (c2).

Additional results for protocol **HighRes** are shown in Supporting Figures S1 and S2. The image results for all shells for a preterm neonate (29 + 0 weeks gestational age) both before and after pre‐processing are illustrated in Supporting Figure S1. Finally, Supporting Figure S2 depicts the results after NODDI 5 analysis for three exemplary subjects. The high resolution allows visualization of the high mean DW signal from the *b* = 2600 shell in the cortex and early‐developing white matter tracts, a pattern that is strikingly different from the adult case. This is of high relevance for the preterm subjects, as the cortical thickness and surface properties have been associated with neurodevelopmental delays 47.

### Acceleration Strategy

The choice of the acceleration parameters was driven by unfolding capacity, SNR, robustness to motion, the EPI bandwidth, and effect on TE of increased RF duration for higher MB factors. The use of all four PEDs forces an EPI echo train length sufficient to encode the longest head dimension, which is AP for transverse slices. Since this is more than required for LR PED, an in plane SENSE factor of 1.2 was used. The choice of this factor was influenced by FOV considerations: The chosen interleaving PED strategy forces a square FOV (145 × 145 mm^2^), which adds about 20% to the required neonatal brain FOV (145 × 120 mm^2^). Therefore, a SENSE factor of 1.2 fits this additionally encoded area well. The choice of the MB factor was driven by g‐factor calculations. These were generated from data acquired on a typical neonate and performing reconstructions for a choice of MB factor and FOV shift, the results for the SENSE factor of 1.2 for both AP‐PA and RL‐LR PEDs. These show, that a relatively low g‐factor can be achieved for MB4, with an optimum for all PEDs if combined with a FOV shift of FOV/3. This result is in accordance with previous studies 48. The obtained numbers are shown in Supporting Table S1.

From here, the design of a slice interleave order fulfilling the required periodic MB slice condition (Eq. (1)) was chosen as *I* = 3 interleaves with a shift of IS = 2.The resulting interleave order for the 16 shot acquisition (*N_e_* = 16) is shown in Figure 2a, with the time of excitation from left to right and the slice dimension from top to bottom, including dotted lines to indicate MB slice pack boundaries. The interleave step *I* is shown in red, the interleave shift IS in black and the distance between respective first and last slices in gray. This is illustrated quantitatively by the slice color in Figure 2 marking the spatial distance between the current and the closest slice excited by the previous RF pulse. Thereby the spatial distance in the optimal pattern (Fig. 2a) equals 3 for any slice, (Fig. 2b) illustrates a suboptimal case with *I* = 3, IS = 1, where the distance varies between 2 (orange) and 5 (green) and (Fig. 2c) a case with I=4,IS=1 where the distance varies between 1 (red) and 6 (green) contributing to increased saturation in neighboring slices and vulnerability to motion.

### Diffusion Preparation

The proportions of diffusion volumes per shell, the maximum *b*‐value 2600, the g‐factor driven choice of the acceleration strategy as well as the limited TA of 20 min set the frame for further optimization to maximize the achievable SNR as stated in Equation (14). Thereby,

1. Step 1: A gradient strength ***G*_max_** between 40 and 80 was selected,
2. Step 2: the corresponding **TE** and—for the fixed TA—**N**_*d*_ determined and finally
3. Step 3: the **SNR** for this acquisition was calculated (Eq. (14)) for each of these settings.

**Figure 6:**
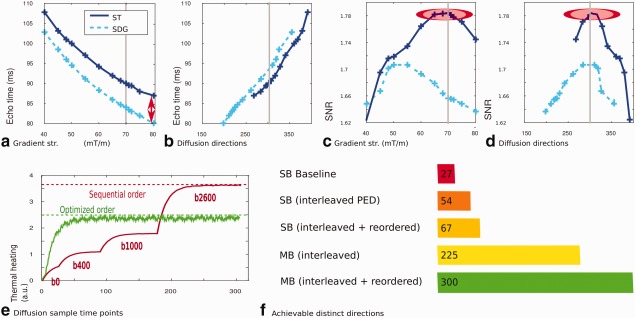
Different optimization steps for the **dHCP** acquisition are shown with the chosen configuration marked with a gray line: (**a**) TE vs. the maximal gradient strength *G*_max_, (**b**) TE vs. number of volumes *N_d_*, (**c**) SNR vs. $G_{\text{max}}$ and (**d**) SNR vs. *N_d_*. The TS extension TS_ext_ for (**e**) sequential ordering with ascending *b*‐values and using the interleaved and optimized ordering. The y‐axis indicated the TS extension factors. (**f**) Improvements in achievable distinct diffusion directions in 20 min.

The results for different combinations of the resulting parameter combinations (*G*_max_, TE, *N_d_*, SNR) are illustrated in Figure 6a–d.

Figure 6a shows the trade‐off between TE vs. *G*_max_ for both ST and SDG and illustrates the decrease in TE achievable using the SDG preparation for all gradient strengths as compared to ST. For example, for $G_{\text{max}}=80\;\text{mT}/m$, TE was lowered from 87 to 80 ms. However, the enhanced load on the gradients with the more demanding SDG preparation leads to a significant decrease in the number of volumes *N_d_* that can be acquired in 20 min. This is evident from Figure 6b where the curve for ST is always to the right of the curve for SDG, so that at any given TE, *N_d_* is larger for the ST case. Contrary to what might be expected, the possible number of directions achieved within 20 min increases as TE increases in both cases. This is because when TE increases, the maximum *b*‐value can be achieved with a lower peak gradient, which decreases the thermal load and so reduces the TS_ext_. The resulting SNR for a 20 min examination calculated using Equation (13), is shown for different gradient strengths in Figure 6c and for different numbers of diffusion directions in Figure 6d, illustrates that under the given parameters, the SDG preparation is not favorable as the benefits achieved by lower TE are counteracted by a significantly reduced number of volumes. An optimal operating point is observed for the ST preparation for a gradient strength of 65–70 mT/m. For the dHCP project 70 mT/m was chosen and led to *N_d_* = 300 (b0:20, b400:64, b1000:88 b2600:128). For this optimization step, an optimal ordering using the strategy described above was assumed. Combining the selected SENSE factor of 1.2 with a half Fourier factor of 0.85 allowed an in‐plane resolution of 1.5 mm^2^ without any penalty in TE or TS_ext_.

### Ordering

Finally, the ordering and PED interleave strategy needs to be determined. The ordering and distribution of the diffusion samples was determined taking uniformity for interrupted scans and thermal simulations into account. Both of these yield optimal temporal orderings, which are of course generally not the same. However, experimentation showed that acquisition orders that are robust against early termination generally interleave all shells, which is favorable for reducing thermal heating of the gradients. Nevertheless, small changes in ordering can substantially impact on the thermal load by avoiding thermal peaks and this reduces duty cycle extensions. Combining these considerations resulted in the following: The four sequential optimization steps which are stated below with the resulting parameter in bold for every step:

1. Generate diffusion sample set consisting of samples (**bval**, **bvec**).
2. Optimality condition: maximal electrostatic repulsion, no ordering.
3. Generate optimal subsets for all required PED's (bval, bvec, **ped**).
4. Optimality condition: maximal electrostatic repulsion within each PED, no ordering.
5. Generate optimal ordering (bval, bvec, ped, **index**).
6. Optimality condition: optimal order of exploration of diffusion encoding space to minimize data loss in the event of curtailed examinations.
7. Generate optimal ordering (bval, bvec, ped, **index**) following Algorithm 2, operating sequentially on chunks of the sample set.
8. Optimality condition: minimal thermal heating).

The bash script given in Supporting Script S3 does steps 1–3. Supporting matlab script S2 serves for step 4. Steps 3 and 4 both modify the index order.

Figure 6e illustrates the evolution of a specific thermal model over time for the sequential and the optimized interleaved ordering. This approach avoids worst case scenarios created by consecutive high *b*‐value directions, which then influence the TR of all 300 volumes. The use of MB allows further optimization, as the number of consecutive excitations with the same gradient load is reduced by the MB factor.

With a MB factor of 4, the influence of thermal reordering of the diffusion samples from a sequential approach with each *b*‐value shell fully acquired before starting on the next, to a fully interleaved approach allowed the acquisition of 300 directions compared to 225. Without MB, reordering allowed the acquisition of 67 directions compared to 54 for the sequential case featuring interleaved PEDs. Assuming the worst‐case scenario – repetition of the same directions in two PEDs, only 27 volumes would be achievable in the given time frame. These improvements are illustrated in Figure 6f.

**Figure 7:**
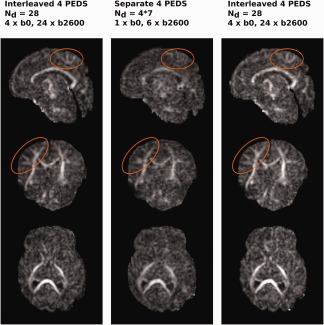
Fractional Anisotropy (FA) maps generated from the interleaved (left column), separate (middle), and repeated interleaved (right) acquisition.

As shown in Figure 3b,c, obtained using the acquisition parameters in protocol **dHCP**, the approach of using all four PEDs rather than repetition of reverse PEDs allows denser angular coverage. The fractional anisotropy maps in Figure 7 depict the results from the illustrative example of the scans of *N_d_* = 28 diffusion samples acquired in one single scan with four interleaved PEDs and 24 collinear directions (left and right columns) and the result obtained from combining for consecutive scans with four PEDs using the same six collinear directions. Regarding distortion correction, the data was processed with a tool originally developed for maximum performance with repeated PEDs 16, but the data is prepared to be processed with future developments using four PEDs. The increased visualization of the anisotropic content is illustrated in the orange circles. When applied to the *N_d_* = 300 directions scheme, the mean nearest neighbor angle decreased from 27.63° (b400), 23.14° (b1000), and 19.11° (b2600) to 19.16°, 16.38°, and 13.5° for the non‐collinear *N_d_* = 300 directions compared to a repetition of 150 directions in two PEDs.

### Comparison to Previously Used dMRI Neonatal Sequence

The achieved protocol was compared to a previously used HARDI protocol on our clinical 3T Philips Achieva on neonates. This protocol needed previously to be acquired in separate scans for different *b*‐values to allow for different diffusion directions tables per *b*‐value. Furthermore, the acquisition of the higher *b*‐value was split in four parts to allow re‐acquisition of subsets if there was data loss due to head motion. The new approach presented here overcomes these limitations and has the advantage of one continuous acquisition, which significantly reduced preparation times, stabilizes the acoustic environment for the infant, and produced more consistent data. In addition, with matched geometry, the conventional protocol takes about 30 min for 96 directions compared to 20 min for 300 directions.

### Motion Robustness

**Figure 8:**
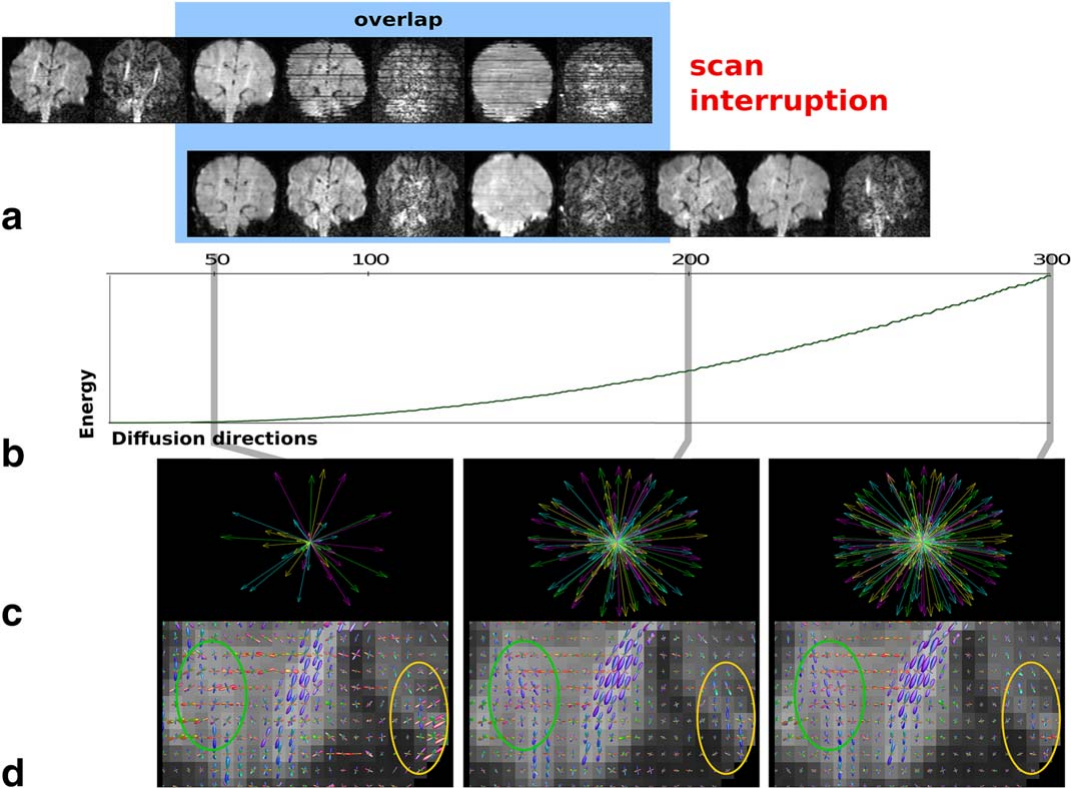
**(a**) Image volumes from the **dHCP** protocol for a subject for whom a restart was required illustrating the overlap and the improved quality of the volumes acquired after resettling and restarting. The use of partially acquired data sets is illustrated in (**b–d**): with the mean bipolar electrostatic repulsion energy shown in (b), the increasing angular coverage after 50, 200, and 300 volumes in (c) and corresponding showing detailed views of the brainstem following a constrained spherical deconvolution analysis of the outer shell generated for the same numbers of sampled directions.

The full dHCP protocol was tested by scanning 400 subjects. Complete dMRI data were obtained on 334 of these: 44 subjects woke up and could not be resettled before the start of the dMRI; 73 woke during the dMRI and of these, 51 were successfully resettled and the scan continued using the restart capacity. A representative case is shown in Figure 8a together with volumes acquired before and after resettling of the baby with an overlap of five volumes. Only 22 scans were aborted, but the uniformly spread diffusion scheme allowed the use of even partially acquired data.

Figure 8b illustrates the increasing bipolar electrostatic repulsion energy for each shell as the acquisition proceeds. Figure 8c illustrates the resulting increasing density as 50, 200, and finally 300 volumes are acquired. The constrained spherical deconvolution (CSD) results from the brain stem in (d), calculated from the outer shell illustrate the growing accuracy in the depiction of crossing fibers (green ellipse) and a general reduction in the background noise (orange ellipse).

### Longer Duration Multishell HARDI (dHCP) Acquisition

**Figure 9:**
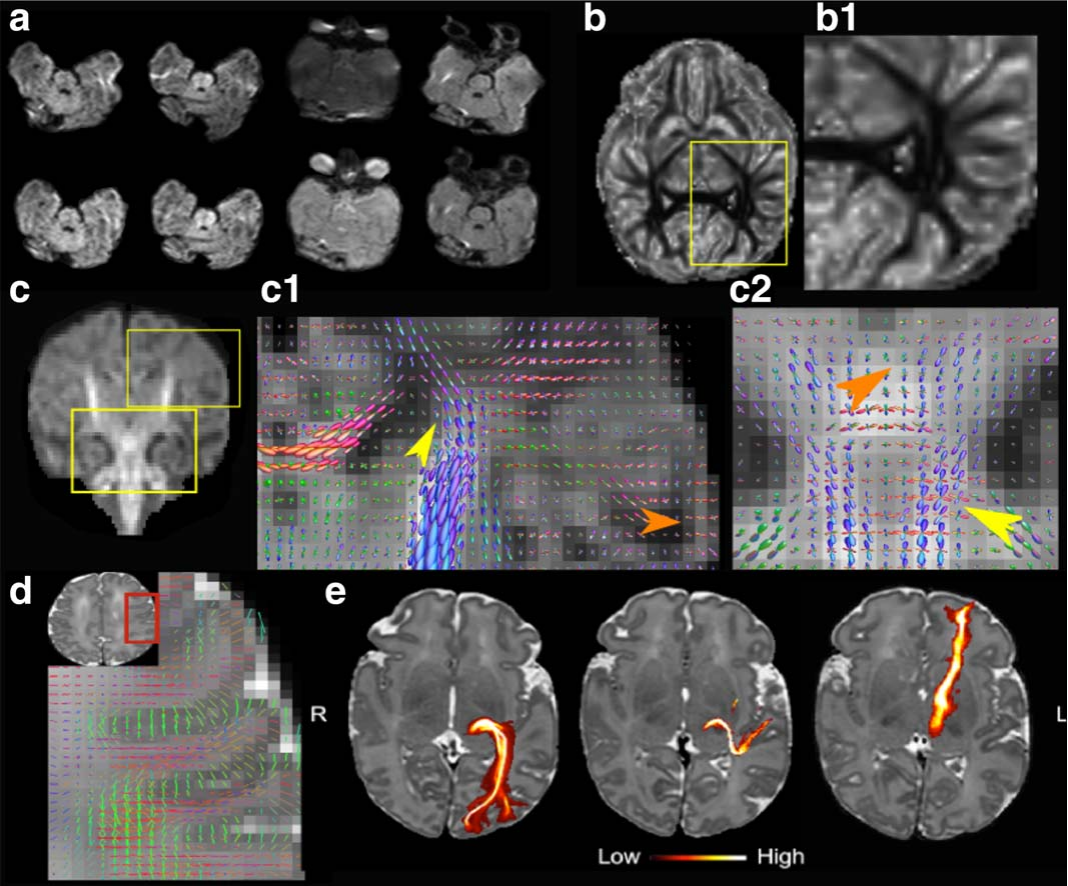
Results from the **dHCP** protocol showing native and distortion corrected images in four exemplary slices (**a**). Analysis results after processing with different algorithms: (**b**) Orientation dispersion index maps obtained using NODDI with a zoom to the white matter (b1) (**c**) Average DWI map obtained with constrained spherical deconvolution (MRTrix3) with two detailed views (c1–c2) and (**d**) using BEDPOSTX. (**e**) Probabilistic tractography results for three subcortical projections.

Reconstructed diffusion weighted images (DWI) and processing results from one exemplary neonate (GA 40 + 2 weeks) for the 19:20 min, 300 volume protocol summarized in Table 1 are shown in Figure 9. An example of slices acquired in all four PEDs at different levels in highly distorted regions is depicted in Figure 9a. These show, that the combined use of all PEDs distributes the regions of collapse and stretching throughout the data so that a more diverse distribution of affected voxels is produced while still allowing effective distortion correction with the given tools (even if the used tools do not reach maximum performance with an interleaved PED scheme). Analyses were performed using constrained spherical deconvolution (MRtrix3) 49, NODDI 5 and model‐based fiber orientation estimation using BEDPOSTX (FSL) 50, 51. The orientation dispersion index maps using NODDI are shown in Figure 9b with a detail view in 9b1. The mean diffusion signal shown in Figure 9c illustrates the inherent low signal in the white matter of the neonatal brain. Higher signal can be observed in areas where myelination is already underway, such as the corticospinal tracts, thus enabling developmental changes to be clearly observed. Two regional detail views (c1‐c2) of the constrained spherical deconvolution results obtained using only the outer shell are discussed in the following. (c1) illustrates the coronal centrum semiovale and shows clear evidence of lateral projections of corpus callosum (red) through corona radiata (blue) and superior longitudinal fasciculus (green, see yellow arrow in figure), as well as the radial orientation of fibers within the cortical ribbon (orange arrow). The coronal detail view of the brainstem in Figure 9c2 clearly demonstrates the crossing fibers in the pons between the cortico‐spinal tracts (blue) and the transverse pontine fibers (red) feeding into middle cerebellar peduncles (green) illustrated by the yellow arrow as well as the crossing pattern of the decussations of the superior cerebellar peduncles near the midline (orange arrow). Figure 9d shows that up to three compartments can be reliably estimated in each voxel using FSL's BEDPOSTX. Crossings in the centrum semiovale, as well as axonal insertions to the cortex are shown in the axial view (right panel). Finally, Figure 9e, showing probabilistic tractography results using FSL's PROBTRACKX 51 for three subcortical projections: the optic radiation, acoustic radiation, and anterior thalamic radiation, indicates that the data quality allows such thin subcortical projections to be tracked. The maximum intensity projections along the axial plane are superimposed on T_2_ images. The colorbar indicates the path probability. Reconstructed images and additional post‐processing results are given in Supporting Figure S3.

## DISCUSSION

Time‐efficiency and flexibility are key requirements for any dMRI acquisition, especially as the complexity and diversity of analysis techniques continue to rise. This article presents a framework combining different developments and modifications to current state‐of‐the art dMRI sequences that together can achieve a highly efficient and optimized acquisition. The approach is able to reveal how different factors can be traded to balance scanner hardware constraints and sequence requirements. It includes complete flexibility in ordering and combining diffusion gradients and EPI phase encode directions (PED), combined with factors to promote efficiency, such as gradient thermal modelling and optimization of *b*‐vector sampling order so that from sequence start there is continued near‐uniformity but growing density in the diffusion domain. A restart capacity was also implemented to accommodate scan interruptions, which allows continuation from the point of interruption with a user defined overlap rather than restarting the entire scan.

The motivation for developing the combined capability was to address the challenge of maximizing the data that can be acquired in the limited TA available during a neonatal examination and at the same time to address the need to be robust to the disruptive effects of motion, scan interruptions, and aborts in neonatal magnetic resonance imaging acquisitions. These were pressing needs for the dHCP, but the same issues arise for many, if not all, clinical studies. Further work might include the automatic identification of motion corrupted volumes and the subsequent automatic choice of overlap or even partial re‐acquisition of corrupted volumes.

The SDG preparation is an interesting alternative to the Stejskal Tanner preparation, yielding significant decreases in TE with associated enhancement in signal (Fig. 4). Initially, this seemed like a gain with no disadvantage except in relation to possible specific requirements on the temporal structure of gradient sensitization that may be needed for some models 5, 6. A further advantage is decreased eddy currents 21, 22 although use of non‐balanced gradients can result in signal loss due to concomitant gradients 52. However, the increased efficiency of SDG is also accompanied by increased demands on the gradient system, so that duty cycle limitations can impose increases in TR to allow cooling. This constraint is increasingly stringent as the number of required diffusion sensitized volumes, and hence total acquisition duration, increases. Further modification of the timings, such as a gap between the first and second diffusion lobe, might allow balancing TE reduction and TR extension better. For the planned 20 min duration of the dHCP diffusion scan, TS extension dominated over reduction in TE leading to a lower overall SNR and scan efficiency with the SGD approach. Optimizing dMRI involves balancing many parameters, such as gradient strength, direction ordering, *b*‐value ratios, thermal extension, and acceleration strategies such as MB, SENSE, and partial Fourier factor. This is a complex multifactorial problem, which we addressed by seeking to optimize SNR for a scan with fixed scan time using models that seek to capture key factors, but inevitably also made simplifications, for example, not including details of slice profiles. The approach has been found to be effective for finding effective choices of sequence parameters, but the SNR prediction should be taken as a guide only. For detailed performance prediction, more sophisticated simulation is required, such as full Bloch simulation taking account of RF pulse choices.

The flexible framework we have presented allows these aspects to be freely combined and provides tools for assessing the effects of different choices. In the case of the dHCP acquisition, an increase in the achievable number of distinct directions from 54 (or even 27 in a theoretical worst‐case scenario) to 300 for a 20 min acquisition was achieved. This allowed for dense angular sampling and also builds in redundancy that allows useful diffusion analysis to proceed when the data is damaged by sporadic motion that may nonetheless have been so intermittent as not to trigger a decision to stop the examination.

The fourfold interleaving of PEDs throughout the scan provides maximal diffusion sampling density compared to repetitions with reversed PED. It provides data suitable for distortion correction methods [such as proposed by 17] that can make use of the available complementary information, acquired in a single scan. Another potential advantage of using all four PEDs is that artefacts, such as from residual fat signals, get dispersed to the maximum number of different locations. As a result, only a quarter of samples for a given voxel are ever likely to be contaminated by a given artefact. The increased information content, diversity and efficiency of the acquisition using interleaved PEDs has been shown here. However, the full benefits for distortion correction will be evident only when combined with an approach which exploits the full range of information. This is the subject of ongoing research.

In view of the tendency of babies to make sporadic movements, it is common in neonatal data for individual slice stacks to be fractured by changes of position, so that realignment is likely to be best addressed at the excited slice pack level rather than at the whole volume level, The use of MB excitation is helpful in this regard as it ensures that multiple slices are acquired from different parts of the brain at the same time. A consequence of realigning individual slices or MB groups of slices is that there is likely to be non‐uniform data within each realigned volume. To decrease the risk of gaps appearing in the data, the slice thickness was chosen to be larger than the target resolution. If the slice centers are located according to the desired target slice separation, resulting in overlapping slices, the target resolution can be partially recovered from the data using super‐resolution algorithms 32, 34 in a post‐acquisition reconstruction stage. The employed parameters for the presented experiments can be found in Table 1.

A future direction of research for the proposed thermal modelling algorithm is the extension from inter‐volume to intra‐volume interleaving of diffusion samples. This could further distribute gradient demand by reducing the number of successive applications of any single gradient waveform below what is needed for a whole volume.

## CONCLUSION

In summary, a design framework for dMRI acquisitions has been presented, together with a strategy to optimize diffusion space sampling accounting for hardware constraints in order to produce dMRI data in a highly efficient and flexible manner. Its development was motivated by the requirements of the dHCP project, and it has enabled a highly efficient acquisition in which a 300 sample multishell diffusion data set can be obtained in 20 min. To illustrate the flexibility of the approach a completely different optimal solution, employing a modified diffusion preparation and a maximal gradient strength for optimized SNR/minimal TE but accepting the resulting prolonged TA due to thermal heating was designed. This is, for the hardware deployed, only feasible for a limited number of high *b* volumes.

Demonstration and use of the combined capability involves a specific implementation on a specific clinical scanner, but all the ingredients, including the choice of interleave pattern, the thermal and motion robustness optimization, and the diffusion preparation developments can have wide applicability, so that the framework can help design dMRI studies more generally.

## Supporting information


**Fig. S1.** Image results in the coronal, sagittal, and tranverse plane from one subject (GA 29 + 0 weeks) scanned using the HighRes protocol. The first row (**a–c**) shows the images after Multiband reconstruction, (**d–f**) after pre‐processing including distortion and motion correction. Thereby, the columns show from left to right data acquired with *b* = 0, *b* = 2600 and *b* = 750. The left column shows a *b* = 0 volume, the middle column a *b* = 2600 volume and the right column a *b* = 750 volume.
**Fig. S2.** NODDI analysis results from three subjects, scanned with the HighRes protocol. First row (**a–c**): preterm neonate GA 29 + 0 weeks, second row (**d–f**): term neonate GA 43 + 2 weeks and third row (**g–i**): term neonate GA 36 + 2 weeks. The orientation dispersion index (OD) is shown in the left, the intra‐cellular volume fraction (Vicf) in the middle and the isotropic volume fraction (Viso) on the right side. The yellow arrow in (b) points toward the cortical surface and the blue arrow in (e) shows the cortical folding depicted in high resolution.
**Fig. S3.** Results from the **dHCP** protocol showing 12 consecutive native images in a mid‐brain slice (**a**). Analysis results after processing with different algorithms: (**c**) Fractional Anisotropy maps, (**d**) ODI maps obtained using NODDI with a zoom to the white matter (d1) (**e**) Average DWI map obtained with CSD (MRTrix3) with two detailed views (e1–e2) and (**f**) using BEDPOSTX. (**g**) Probabilistic tractography results for three subcortical projections.
**Table S1**. g‐Factor Calculations for Different Combinations of Multiband Factor, Shift Factor and Phase Encoding Direction.
**Table S2**. Symbols Used in the Slice Order Optimization Section.
**Table S3**. Symbols Used in the Thermal Heating Section.
**Table S4**. Symbols Used in the Diffusion Optimization Section.
**Script S1**. Slice order optimization.
**Script S2**. Thermal modelling optimization.
**Script S3**. Generation of evenly distributed multi‐shell 4‐PED diffusion samples.

## Footnotes

1 *http://www.developingconnectome.org/.
