# Supplementary material for: Time‐efficient and flexible design of optimized multishell HARDI diffusion
Source: Magn Reson Med. 2017 May 30;79(3):1276–92. doi: 10.1002/mrm.26765 (PMC5811841; doi:10.1002/mrm.26765)
Supplement: Supplementary file 1 — Fig. S1. Image results in the coronal, sagittal, and tranverse plane from one subject (GA 29 + 0 weeks) scanned using the HighRes protocol. The first row (a–c) shows the images after Multiband reconstruction, (d–f) after pre‐processing including distortion and motion correction. Thereby, the columns show from left to right data acquired with b = 0, b = 2600 and b = 750. The left column shows a b = 0 volume, the middle column a b = 2600 volume and the right column a b = 750 volume. Fig. S2. NODDI analysis results from three subjects, scanned with the HighRes protocol. First row (a–c): preterm neonate GA 29 + 0 weeks, second row (d–f): term neonate GA 43 + 2 weeks and third row (g–i): term neonate GA 36 + 2 weeks. The orientation dispersion index (OD) is shown in the left, the intra‐cellular volume fraction (Vicf) in the middle and the isotropic volume fraction (Viso) on the right side. The yellow arrow in (b) points toward the cortical surface and the blue arrow in (e) shows the cortical folding depicted in high resolution. Fig. S3. Results from the dHCP protocol showing 12 consecutive native images in a mid‐brain slice (a). Analysis results after processing with different algorithms: (c) Fractional Anisotropy maps, (d) ODI maps obtained using NODDI with a zoom to the white matter (d1) (e) Average DWI map obtained with CSD (MRTrix3) with two detailed views (e1–e2) and (f) using BEDPOSTX. (g) Probabilistic tractography results for three subcortical projections. Table S1. g‐Factor Calculations for Different Combinations of Multiband Factor, Shift Factor and Phase Encoding Direction. Table S2. Symbols Used in the Slice Order Optimization Section. Table S3. Symbols Used in the Thermal Heating Section. Table S4. Symbols Used in the Diffusion Optimization Section. Script S1. Slice order optimization. Script S2. Thermal modelling optimization. Script S3. Generation of evenly distributed multi‐shell 4‐PED diffusion samples. [file MRM-79-1276-s001.pdf]

Additional Material for *"Time-efficient and flexible design of optimised multi-shell HARDI diffusion"*

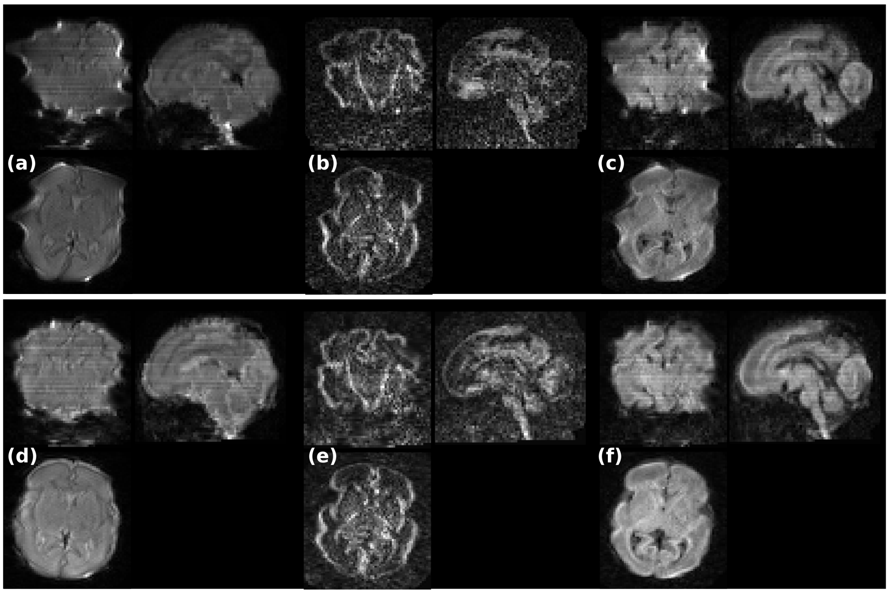

Supporting Figure S1: Image results in the coronal, sagital and tranverse plane from one subject (GA 29+0 weeks) scanned using the **HighRes** protocol. The first row (a-c) shows the images after Multiband reconstruction, (d-f) after pre-processing including distortion and motion correction. The left column shows a b=0 volume, the middle column a b=2600 volume and the right column a b=750 volume.

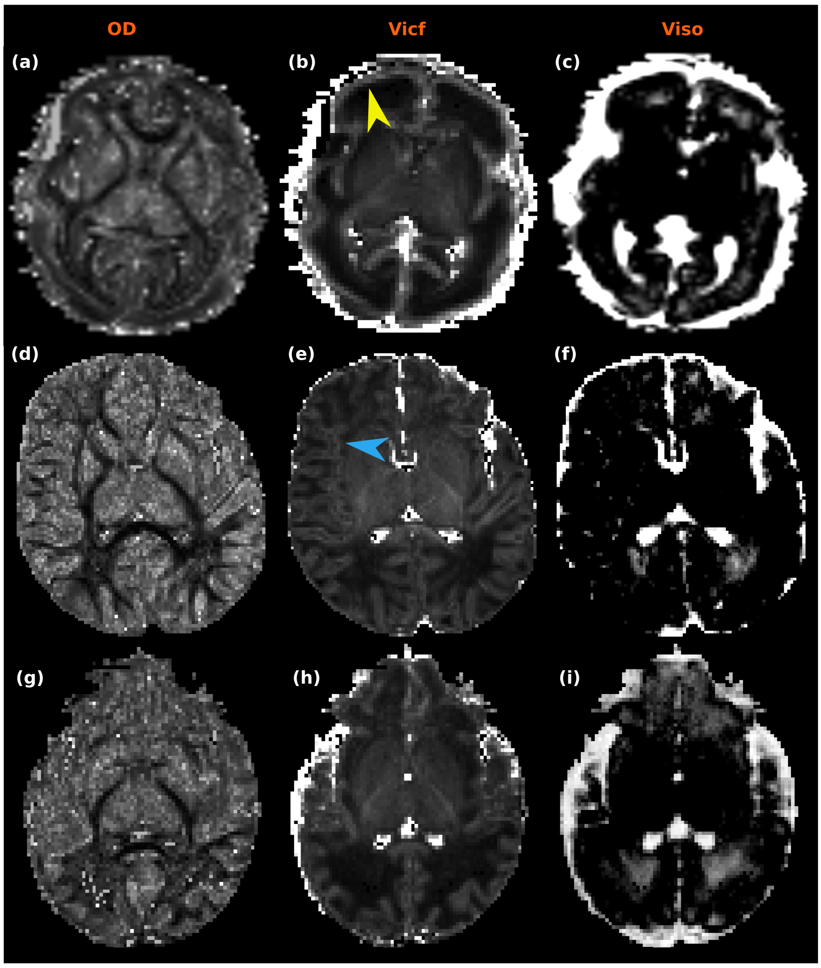

Supporting Figure S2: NODDI analysis results from three subjects, scanned with the HighRes protocol. First row (a-c): preterm neonate GA 29+0 weeks, second row (d-f): term neonate GA 43+2 weeks and third row (g-i): term neonate GA 36+2 weeks.
The orientation dispersion index (OD) is shown in the left, the intra-cellular volume fraction (Vicf) in the middle and the isotropic volume fraction (Viso) on the right side.
The yellow arrow in (b) points towards the cortical surface and the blue arrow in (e) shows the cortical folding depicted in high resolution.

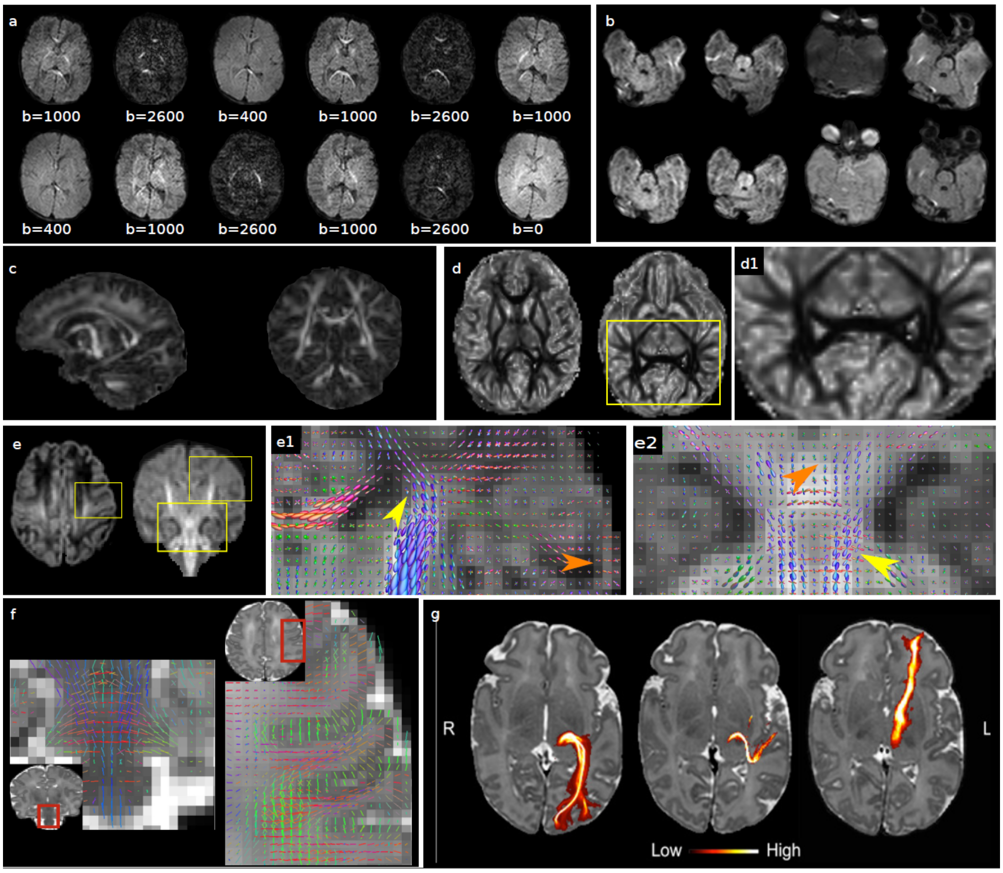

Supporting Figure S3: Results from the dHCP protocol showing 12 consecutive native images in a mid-brain slice (a). Analysis results after processing with different algorithms: (c) Fractional Anisotropy maps, (d) ODI maps obtained using NODDI with a zoom to the white matter (d1) (e) Average DWI map obtained with CSD (MRTrix3) with two detailed views (e1-e2) and (f) using BEDPOSTX. (g) Probabilistic tractography results for three subcortical projections.

	LR/RL	MB2	MB3	MB4	MB5	MB6	AP/PA	MB2	MB3	MB4	MB5	MB6
Shift 2		1.12	1.46	2.13	4.19	8.34		1.13	1.49	2.17	4.14	8.09
Shift 3		1.14	1.49	2.04	3.42	5.67		1.15	1.53	2.09	3.40	5.56
Shift 4		1.17	1.62	2.25	3.64	5.92		1.18	1.66	2.35	3.79	5.61

Supporting Table S1: g-factor calculations for different combinations of Multiband factor, shift factor and phase encoding direction.

	**Description**	unit
N_e	Number of excitations	n/a
N_s	Number of slices	n/a
N_m	Multiband factor	n/a
I	Interleave step	*slices*
IS	Interleave shift	*slices*
s_i	Start slice in interleave i	*slice*
i	Interleave index	-
δ	Length diffusion gradient ST	[ms]
Δ	Diffusion time ST	[ms]

Supporting Table S2: Symbols used in the slice order optimization section

Supporting Table S2: Symbols used in the slice order optimization section.

**Symbol**	**Description**	**unit**
d_j	Diffusion sample description at TR index j	n/a
j	TR index	-
i	Direction index	-
m	Time segment index	-
t_m	Segment length	[ms]
P_i^m	Dissipated Power	[VA]
G_i^m	Gradient strength for axis i and time index m	[mT/m]
H_i	Hardware constant	$[VAm^2/T^2]$
L_i^m	Combined thermal load at time index m	[VA]
L_i^j	Combined thermal load at diffusion index j	[VA]
y	Loop index for the optimization algorithm	-
L_{max}^y	Combined thermal load at diffusion index j	[VA]
L_{Hmax}	Combined thermal load at diffusion index j	[VA]
L	Maximally obtained load	[VA]
τ	Time constant	[ms]
D^j	Available diffusion samples at TR index j	n/a
Π^j	Permutation of diffusion samples at TR index j	n/a
n	Index for the diffusion samples in D^j	-

Supporting Table S3: Symbols used in the thermal heating section

Supporting Table S3: Symbols used in the thermal heating section.

	**Description**	unit
$G_{\max}$	Maximal gradient strength	[mT/m]
σ	Slew rate	[mT/m/s]
δ	Length diffusion gradient ST	[ms]
Δ	Diffusion time ST	[ms]
$\mathbf{d}$	Diffusion parameters	n/a
$\mathbf{h}$	Hardware factor	n/a
r	Ratio between σ_2 and σ_3	-
t_{ex}	Length of the excitation pulse	[ms]
t_{re}	Length of the refocusing pulse	[ms]
t_{pre}	Time between ex. and re. pulse	[ms]
t_{post}	Time between re. pulse and read-out	[ms]
G	Gradient strength	[mT/m]
G_{over}	Gradient strength $> G_{max}$	[mT/m]
$\tilde{b}$	Maximal achievable b-value	$[s/mm^2]$
b	Prescribed b-value	$[s/mm^2]$
t_{ex0}	Ex. pulse start time	[ms]
t_{re0}	Re. pulse start time	[ms]

Supporting Table S4: Symbols used in the diffusion optimization section

Supporting Table S4: Symbols used in the diffusion optimization section.

<u>Supporting Script S1</u>: Slice order optimization

Programming language: matlab

Usage: Parameters Ne, I, IS within script

```
%%%%reviewer parameters
Ne=5;   %number of excitations per slice pack
I=3;    %interleave
IS=1;   %interleave shift


maxshots= I+2;
dist_first=zeros(maxshots,1);
dist_last=zeros(maxshots,1);
slices=zeros(Ne,shots);


for s=1:maxshots
    dist_first(s) = mod((s-1)*IS,I);
    dist_last(s) = mod(Ne-(dist_first(s)+1),I);
    slices(dist_first(s)+1,s)=s;
    slices(Ne-dist_last(s),s)=s;
end
dist_first'
dist_last'
distances=dist_first(2:end)+dist_last(1:end-1)


subplot(1,2,1)
imagesc(slices);
xlabel('interleave number');            ylabel('slices in slice pack 1');         title('First and last slice');
subplot(1,2,2)
if max(distances)~=min(distances)
    plot(distances,'r+-','linewidth',3)
else
    plot(distances,'g+-','linewidth',3)
end
xlabel('interleave number');            ylabel('distance first to last from previous interleave');        title('Distances');
```

Supporting Script S2*: Thermal modelling optimization*

Programming language: matlab

Usage: constructOrder(grad, level, strength, name)

Example: constructOrder(dw.txt, 1.1, 80, dw_opt.txt)

```
function[gradOrder]= constructOrderTemplate(grad,level,strength,name,opt)
global PC   global levelStep
levelStep=0.1;
settings.seq.tk=6.6;
settings.hw.therm=[100];
settings.hw.Nmodel=1;
settings.hw.HmaxLoad = [100];
settings.hw.sens=[20,20,20];
settings.hw.res=[0.05 0.05 0.05];
settings.seq.Gmax = 80;
settings.seq.mid=0.41
settings.seq.time=95;
settings.seq.d2=11;
settings.seq.r=1.3;


[Ng,dirs,rm,L,Lmax,Lmaxa,PC]=dcEvalTemplate(grad,strength,settings);
    if (opt==true)
        L=zeros(Ng+1,settings.hw.Nmodel,3);
        Lmax=zeros(Ng+1,settings.hw.Nmodel,3);
        Lmaxa=zeros(Ng+1,settings.hw.Nmodel);
        dirsNew=zeros(size(dirs));
        dirOrder=zeros(Ng,1);


        valid=false;
        while valid==false
            grad(:,6)=0; %not used yet
            grad(1,6)=1; %used yet
            count=true;


            for y=2:Ng
                    Lcur=(L(y,:,:)); %eval current PC
                    [index,res]=findDirection(Lcur,grad,PC,level,settings);
                    if (res==false)
                        count=false;
                        valid=false;
                        break;
                    end
                    if index==-1
                        index=y;
                    end
                    dirsNew(y,:)=dirs(index,1:3);
                    dirOrder(y)=index;
                    grad(index,6)=1;


                    L(y+1,:,:)=calculateLoadsTemplate((L(y,:,:)),(PC(index,:)),settings);


                    for l=1:settings.hw.Nmodel
                    for d=1:3
                          Lmax(y+1,l,d)=max(Lmax(y,l,d),L(y+1,l,d));
                          Lmaxa(y+1,l)=max(Lmaxa(y+1,l),Lmax(y+1,l,d));
                    end
                    end
            end
            if count==true
                valid=true;
            end
            level=level+levelStep;
        end
        dirOrderFree=dirOrder(2:end);
        dlmwrite(name,dirOrderFree,' ')


         for mod=1:settings.hw.Nmodel
        Lmax(:,mod,:)=Lmax(:,mod,:)./HmaxLoad(mod);
        L(:,mod,:)=L(:,mod,:)./HmaxLoad(mod);
        end
        figure(2)
        subplot(3,1,1)
        plot(squeeze(L(:,1:settings.hw.Nmodel,1)),'linewidth',2)
        legend('1ch model x')        hold on
        plot(squeeze(Lmax(:,1:settings.hw.Nmodel,1)),'--')
        subplot(3,1,2)
        plot(squeeze(L(:,1:settings.hw.Nmodel,2)),'linewidth',2)
        legend('1ch model y')        hold on
        plot(squeeze(Lmax(:,1:settings.hw.Nmodel,2)),'--')
        subplot(3,1,3)
```

```
        plot(squeeze(L(:,1:settings.hw.Nmodel,3)),'linewidth',2)
        legend('1ch model z')        hold on
        plot(squeeze(Lmax(:,1:settings.hw.Nmodel,3)),'--')


    end
end
function[index,res]=findDirection(Lcur,grad,PC,fixed,settings)
iPC=PC;
global levelStep;


for index=1:size(grad,1)
    Ltry(index,:,:)=calculateLoadsTemplate((Lcur),squeeze(iPC(index,:)),settings);
end
for mod=1:settings.hw.Nmodel
   Ltry(:,mod,:)=Ltry(:,mod,:)./settings.hw.HmaxLoad(mod);
end
possible=zeros(size(grad,1),1);
fixedtmp=fixed;
while(sum(possible)==0)
    for i=1:size(grad,1)
        if (grad(i,6)==0)
            possible(i)=max(max((Ltry(i,:,:))))<fixed;
        end
    end
    fixed=fixed+levelStep;
end
res=true;
if abs(fixed-fixedtmp)>(levelStep*2)
    res=false;
end
[maPT,inPT]=max(iPC);


iPC(:,1)=iPC(:,1).*possible;
iPC(:,2)=iPC(:,2).*possible;
iPC(:,3)=iPC(:,3).*possible;
[maPTh,inPTh]=max(iPC);
[maPTh,inPThh]=max(maPTh);
inPT=inPTh(inPThh);
index=inPT;


end


function[Ng,dirs,rm,L,Lmax,Lmaxa,PC]=dcEvalTemplate(grad,G,settings)
Ng=size(grad,1);
L=zeros(Ng+1,settings.hw.Nmodel,3);
Lmax=zeros(Ng+1,settings.hw.Nmodel,3);
Lmaxa=zeros(Ng+1,settings.hw.Nmodel);


for y=1:Ng
    dir=grad(y,1:3);
    dir=dir./norm(dir);
    ped=grad(y,5);
    bval=grad(y,4);


    dir=dir.*sqrt(bval/settings.seq.mid)*(G/settings.seq.Gmax);
    dirs(y,:)=dir;
    [rm(y,:)]=rms(settings.seq.d2,settings.seq.r,dir,ped,settings.seq.time);


        for d=1:3
            PC(y,d)=(rm(y,d).^2*settings.hw.sens(d).^2*settings.hw.res(d))/(1000);
        end


        L(y+1,:,:)=calculateLoadsTemplate((L(y,:,:)),(PC(y,:)),settings);
        for l=1:settings.hw.Nmodel
        for d=1:3
              Lmax(y+1,l,d)=max(Lmax(y,l,d),L(y+1,l,d));
              Lmaxa(y+1,l)=max(Lmaxa(y+1,l),Lmax(y+1,l,d));
        end
        end
end
for mod=1:settings.hw.Nmodel
    Lmax(:,mod,:)=Lmax(:,mod,:)./settings.hw.HmaxLoad(mod);
    L(:,mod,:)=L(:,mod,:)./settings.hw.HmaxLoad(mod);
end


        subplot(3,1,1)
        plot(squeeze(L(:,1:settings.hw.Nmodel,1)),'linewidth',2)
        legend('1ch model x')  hold on
        plot(squeeze(Lmax(:,1:settings.hw.Nmodel,1)),'--')
        subplot(3,1,2)
        plot(squeeze(L(:,1:settings.hw.Nmodel,2)),'linewidth',2)
        legend('1ch model y')  hold on
        plot(squeeze(Lmax(:,1:settings.hw.Nmodel,2)),'--')
```

```
        subplot(3,1,3)
        plot(squeeze(L(:,1:settings.hw.Nmodel,3)),'linewidth',2)
        legend('1ch model z')  hold on
        plot(squeeze(Lmax(:,1:settings.hw.Nmodel,3)),'--')
end


function[tmpL]=calculateLoadsTemplate(Lold,PCt,settings)
    tmpL=zeros(settings.hw.Nmodel,3);
    l=1; %1 model
    decay=exp(-settings.seq.tk/settings.hw.therm(l));
    for d=1:3
        tmpL(l,d)=decay*Lold(l,d)+(1-decay)*PCt(l,d);
    end
end
```

Supporting Script S3: *Generation of evenly distributed multi-shell 4-PED diffusion samples*

Programming language: bash script

Usage: gen_scheme numPE [bvalue ndir]

Example: ./gen_scheme 4 0 10 750 15 2600 30

```
set -e
if [ "$#" -eq 0 ]; then
    echo "usage: gen_scheme numPE [ bvalue ndir ]..."
    exit 1
else
  nPE=$1
  if [ $nPE -ne 1 ] && [ $nPE -ne 2 ] && [ $nPE -ne 4 ]; then
    echo "ERROR: numPE should be one of 1, 2, 4"
    exit 1
  fi
  shift
  # store args for re-use:
  ARGS="$*"

  # print parsed info for sanity-checking:
  echo "generating scheme with $nPE phase-encode directions, with:"
  while [ ! -z "$1" ]; do
    echo "    b = $1: $2 directions"
    shift 2
  done

  perm="1" #"-perm 1000"
  # reset args:
 # set -- "${ARGS[@]}"
  set -- $ARGS
 merge=""
  while [ ! -z "$1" ]; do
    echo "====================================="
    echo "generating directions for b = $1..."
    echo "====================================="

    merge=$merge" "$1
set -x
    dirgen $2 dirs-b$1-$2.txt -force
    if [ $nPE -gt 1 ]; then
      dirsplit dirs-b$1-$2.txt dirs-b$1-$2-{1..2}.txt -force $perm
      if [ $nPE -gt 2 ]; then
        dirsplit dirs-b$1-$2-1.txt dirs-b$1-$2-1{1..2}.txt -force $perm
        dirsplit dirs-b$1-$2-2.txt dirs-b$1-$2-2{1..2}.txt -force $perm
                # TODO: the rest...
                for n in dirs-b$1-$2-{1,2}{1,2}.txt; do
          dirflip $n ${n%.txt}-flip.txt -force $perm
                  merge=$merge" "${n%.txt}-flip.txt
        done
      else
        for n in dirs-b$1-$2-{1,2}.txt; do
          dirflip $n ${n%.txt}-flip.txt -force $perm
          merge=$merge" "${n%.txt}-flip.txt
                done
      fi
    else
      dirflip dirs-b$1-$2.txt dirs-b$1-$2-flip.txt -force $perm
      merge=$merge" "dirs-b$1-$2-flip.txt
    fi
    shift 2
  done
  echo $merge
  dirmerge $nPE $merge dw_scheme.txt -force
fi
```
